# Research on High-Resolution Miniaturized MEMS Accelerometer Interface ASIC

**DOI:** 10.3390/s20247280

**Published:** 2020-12-18

**Authors:** Xiangyu Li, Yangong Zheng, Xiangyan Kong, Yupeng Liu, Danling Tang

**Affiliations:** 1 Faculty of Electrical Engineering and Computer Science, Ningbo University, Ningbo 315211, China; lixiangyu@nbu.edu.cn (X.L.); zhengyangong@nbu.edu.cn (Y.Z.); 2 Southern Marine Science and Engineering Guangdong Laboratory, Guangdong Key Laboratory of Ocean Remote Sensing (LORS), South China Sea Institute of Oceanology, Chinese Academy of Sciences, Guangzhou 510301, China; liuyupeng@scsio.ac.cn (Y.L.); lingzistdl@126.com (D.T.)

**Keywords:** MEMS accelerometers, interface circuit, PID feedback, closed-loop

## Abstract

High-precision microelectromechanical system (MEMS) accelerometers have wide application in the military and civil fields. The closed-loop microaccelerometer interface circuit with switched capacitor topology has a high signal-to-noise ratio, wide bandwidth, good linearity, and easy implementation in complementary metal oxide semiconductor (CMOS) process. Aiming at the urgent need for high-precision MEMS accelerometers in geophones, we carried out relevant research on high-performance closed-loop application specific integrated circuit (ASIC) chips. According to the characteristics of the performance parameters and output signal of MEMS accelerometers used in geophones, a high-precision closed-loop interface ASIC chip based on electrostatic time-multiplexing feedback technology and proportion integration differentiation (PID) feedback control technology was designed and implemented. The interface circuit consisted of a low-noise charge-sensitive amplifier (CSA), a sampling and holding circuit, and a PID feedback circuit. We analyzed and optimized the noise characteristics of the interface circuit and used a capacitance compensation array method to eliminate misalignment of the sensitive element. The correlated double sampling (CDS) technology was used to eliminate low-frequency noise and offset of the interface circuit. The layout design and engineering batch chip were fabricated by a standard 0.35 μm CMOS process. The active area of the chip was 3.2 mm × 3 mm. We tested the performance of the accelerometer system with the following conditions: power dissipation of 7.7 mW with a 5 V power supply and noise density less than 0.5 μg/Hz^1/2^. The accelerometers had a sensitivity of 1.2 V/g and an input range of ±1.2 g. The nonlinearity was 0.15%, and the bias instability was about 50 μg.

## 1. Introduction

Recently, microintelligent sensors have seen a wide range of market demand [1,2]. Capacitive microelectromechanical system (MEMS) accelerometers as inertial sensors are widely used in GPS-aided navigators in the consumer market [3,4,5]. Capacitive MEMS accelerometers have the advantages of low power consumption, miniaturization, good stability, and easy integration with the CMOS process [6,7,8,9]. High-performance capacitive accelerometers with an accuracy of sub-μg level occupy a large market share in inertial navigation. MEMS capacitive accelerometers have two working modes: open-loop and closed-loop. The structure of a microaccelerometer with open-loop output is simple, but the signal bandwidth is limited by the sensitive element structure and the signal input range is greatly reduced [10,11,12,13]. Closed-loop accelerometers are applied by electrostatic servo control, which can improve the linearity to a great extent. Because the open-loop structure needs to achieve high sensitivity, the signal-to-noise ratio (SNR) of microaccelerometers can be improved. In [14], the researchers proposed a high-order closed-loop system for a high-sensitivity low-Q accelerometer, which resulted in an inherently stable system. However, the measured closed-loop system did not achieve sub-μg equivalent resolution. In [15,16], a sub-μg equivalent resolution continuous-time capacitive accelerometer interface using a high-Q mechanical element was reported. The researchers used a high-resolution analog-to-digital converter (ADC) to digitize the feedback signal in order to achieve a high-resolution digital output. The use of ADC deteriorated the noise floor by 6 dB but obviously increased the power consumption. In [17], the researchers proposed a detection methodology for paralleling parasitic capacitance based on a harmonic distortion self-test. The result showed that a detecting resolution better than −120 dB could be achieved. After calibration, the parasitic mismatch-induced bias could be suppressed to sub-mg level. With the development of MEMS technology, the integration, intelligence, and digitization of capacitive MEMS accelerometers have become an important research direction [18,19,20]. It is of great significance to study closed-loop interface circuits for high-performance capacitive MEMS accelerometers. Misalignment of the accelerometer sensitive element and system noise are the key parameters that determine the performance of accelerometers. High-performance accelerometers have strict requirements on output signal distortion, and an electrostatic feedback method is generally used to form a closed-loop control system. Analysis and optimization of the noise parameters of accelerometers need further research.

In this study, we analyzed the main source of noise in a closed-loop interface circuit and used a switched capacitor interface circuit with an electrostatic time-multiplexing feedback technique. The sensitive element and electrostatic feedback were separated in time sequence using the same electrode. The interface circuit provided a proportion integration differentiation (PID) feedback control mechanism in the loop to improve system stability. We used correlated double sampling (CDS) in the sample and hold circuit to eliminate interface mismatch and insufficient loop gain. Microaccelerometers with sub-μg precision output have a lot of application requirements in the field of geophones. We analyzed and optimized the noise characteristics of the interface circuit. The gain booster technique was applied in the charge-sensitive amplifier (CSA) and PID feedback circuit to eliminate deterioration of the overall noise floor. Therefore, the noise theory analysis, electrostatic time-multiplexing technology, and PID feedback control mechanism of high-precision closed-loop microaccelerometers were mainly studied in this work.

The rest of the paper is organized as follows. In Section 2, the design of the accelerometer sensitive element, capacitance compensation array, front-end charge-sensing circuit, sample and hold circuit, and PID feedback control circuit are introduced. In Section 3, we show a detailed analysis and optimization based on the noise characteristics and sequential control of microaccelerometers with ASIC interface circuit. Finally, Section 4 concludes our study of MEMS accelerometers with high-precision integrated circuit by discussing the test results, which showed that the performance of our microaccelerometer has great advantages in terms of noise floor and power dissipation compared to previously reported works.

## 2. Materials and Methods

### 2.1. Accelerometer Sensitive Element and High-Resolution Closed-Loop Interface Circuit

**Figure 1:**
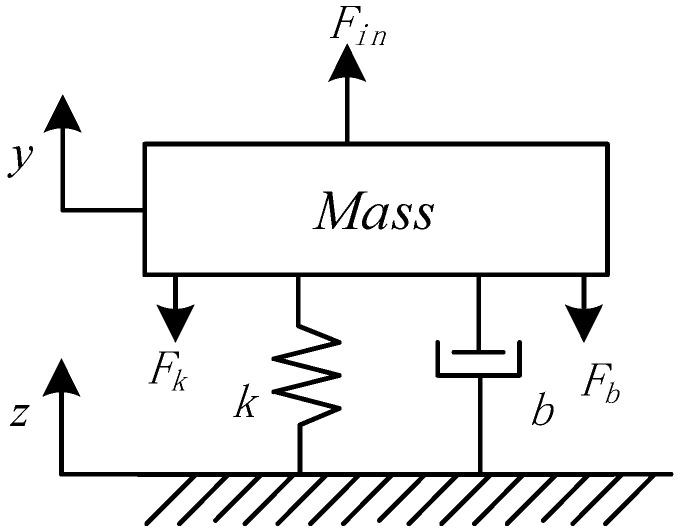
Equivalent schematic of the mechanical structure of a microaccelerometer.

In order to achieve high-resolution accelerometers, a low-noise sensor element and an interface circuit are critical. We used a high-resolution sensitive element with an extremely low Brownian noise floor. The mechanical structure of a microaccelerometer mainly includes a mass block, a cantilever beam, and a fixed plate. The mechanical structure of the sensitive element is shown in Figure 1. When an external acceleration signal is applied to the sensitive element, the mass is affected by elastic force *F_in_* and damping force *F_b_*. The accelerometer system moves the displacement *z*, and the mass block moves the displacement *y*. Then, the relative displacement *x* produced by the mass block is equal to *y − z*. As shown in Figure 1, *m* is the mass of the mass block, *k* is the elastic coefficient, and *b* is the damping coefficient. The equivalent mechanical structure diagram can be regarded as a second-order vibration system composed of mass, spring, and damping [21].

According to Newton’s second law, the mechanical equation can be written as follows:

(1) $$m\frac{d^{2}y}{dt^{2}}+b\frac{d(y-z)}{dt}+k(y-z)=0$$

We put *x = y − z* into the above Equation (1):

(2) $$m\frac{d^{2}x}{dt^{2}}+b\frac{dx}{dt}+kx=-m\frac{d^{2}z}{dt^{2}}=-ma$$

We can obtain the transfer function from acceleration to displacement of an accelerometer by Laplace transformation of Equation (2):

(3) $$H_{ms}(s)=\frac{x(s)}{a_{in}(s)}=\frac{1}{s^{2}+\frac{b}{m}s+\frac{k}{m}}=\frac{1}{s^{2}+\frac{w_{0}}{Q}s+w_{0}^{2}}$$

In Equation (3), $w_{o}$ is the resonant frequency of the mechanical structure of an accelerometer, and *Q* is its quality factor.

(4) $$w_{o}=\sqrt{\frac{k}{m}}$$

(5) $$Q=\frac{w_{0}m}{b}$$

The scale factor of the sensitive structure can be obtained from Equation (3). Its physical meaning is the displacement of the mass block when the unit acceleration is input. We can use $\left|\frac{X(\omega )}{a(\omega )}\right|$ to express this:

(6) $$\left|\frac{X(\omega )}{a(\omega )}\right|=\{\begin{array}{cc} \frac{1}{\omega_{0}^{2}} & \omega <<\omega_{0}\\ \frac{Q}{\omega_{0}^{2}} & \omega \approx \omega_{0}\\ \frac{1}{\omega^{2}} & \omega >>\omega_{0} \end{array}$$

It can be seen that when the input signal frequency band is far less than the resonance frequency of the mechanical structure, the relationship between the relative displacement of the mass block and the input acceleration signal is approximately linear. The sensitivity is only inversely proportional to the square of the resonant frequency, while it is nonlinear in other frequency bands. We can use this linear relationship to indirectly measure the input acceleration signal. High-precision accelerometers always work in a closed-loop state in order for the moving mass to be controlled in a small displacement range. Accelerometers with a closed-loop structure have wide bandwidth and dynamic range (DR), high linearity, and good stability.

**Figure 2:**
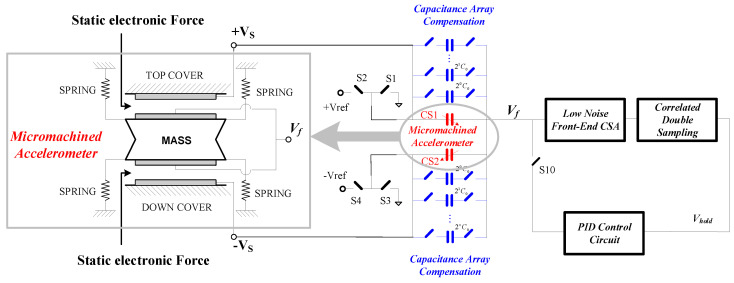
System architecture of a closed-loop microaccelerometer.

**Figure 3:**
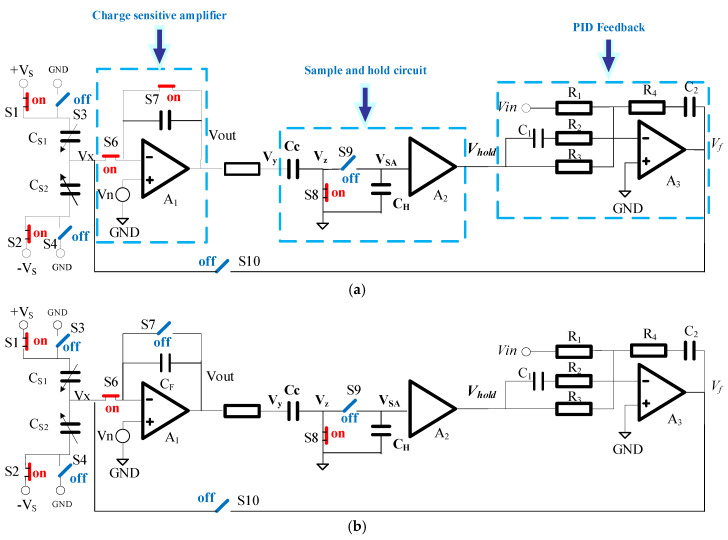

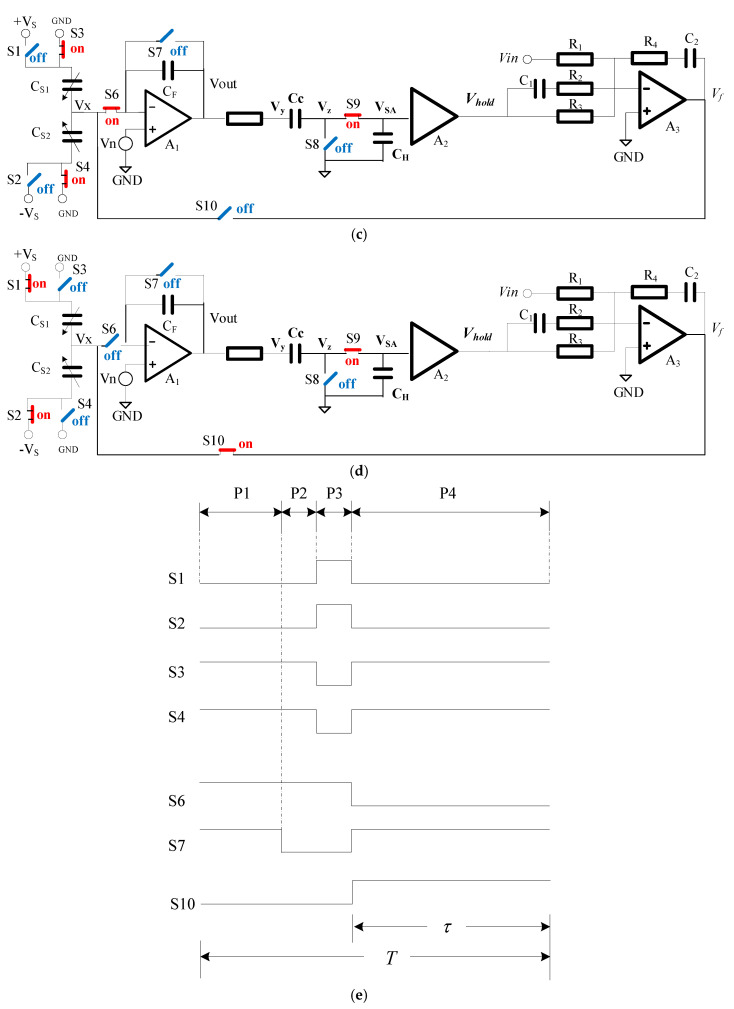
Switched capacitor topology diagram of closed-loop interface circuit: (**a**) the amplifier error pickup phase (P1); (**b**) the charge amplifier preparation phase (P2); (**c**) the charge sampling phase (P3); (**d**) the electrostatic closed-loop feedback phase (P4); (**e**) the schematic of main switching phase in ASIC chip.

In the traditional scheme, the output voltage of the microaccelerometer is fed back to the input of the charge-sensitive amplifier through the filter [22,23]. The mass block voltage of the micromechanical element is changed by the operational amplifier so as to achieve the purpose of electrostatic force feedback. The disadvantage of this scheme is that the feedback voltage frequency should be separated from the modulation frequency (1 MHz) in order to reduce the coupling of the closed-loop feedback voltage to the capacitance change pick-up circuit. This requires the bandwidth of the front-stage charge amplifier to be in the order of tens of megahertz. The adopted high-pass filter will lead to a change in the signal phase and the sensor demodulation phase. This leads to the drift of zero output and scale factor of microaccelerometers. At the same time, the common-mode input voltage range of the charge amplifier limits the dynamic output range of microaccelerometers. This work proposes a novel time-multiplexing feedback of the electrostatic force method. In this system, the charge-sensitive time sequence is separated from the electrostatic feedback time sequence so as to avoid coupling of the electrostatic feedback voltage to the charge detection path and improve the reliability of accelerometers. The schematic diagram of the system is shown in Figure 2. The microaccelerometer interface ASIC chip controls the turn-on and turn-off of analog switches S6 and S10 by a sequential circuit. The circuit diagram with a timing switch is shown in Figure 3. The capacitance change detection phase and electrostatic force feedback phase are separated in time domain so as to minimize the feedthrough and coupling between the feedback signal and the detection signal. At the same time, we used the correlated double sampling technique in the charge-sensitive amplifier circuit and noise optimization so that the interface circuit can be realized at a dynamic range of 120 dB.

Because the modulated frequency is the same as the clock signal, misalignment of the sensitive element cannot be eliminated by AC coupling and DC feedback. Sensor misalignment can be eliminated by designing an electrostatic actuator to pull the mass block back to the central position in the mechanical structure. However, sensor misalignment can be large, and large electrostatic actuators and high voltage are required. We used a capacitance compensation array method to eliminate sensor misalignment. The actual equivalent circuit considering parasitic capacitance of a capacitive microaccelerometer is shown in Figure 2 with blue and red marks. When there is no input acceleration signal, the switch is off. The equivalent capacitance satisfies the following equation:

(7) $$\{\begin{array}{l} C_{S1}=C_{S2}\\ C_{P1}-C_{P2}=C_{P}\neq 0 \end{array}$$

In Equation (7), *C_S_* is the sensitive capacitance, and *C_P_* is the parasitic capacitance. The equivalent circuit of the MEMS structure of a microaccelerometer is shown in Figure 2. The output of the capacitance-sensitive detection circuit produces offset voltage. Because the feedback path is carried out by a PID circuit, the integrator will force the voltage node, *V_hold_* = 0. The electrostatic feedback will force the mass block position to change Δ*d*. When Δ*d* is very small, this satisfies the following equation:

(8) $$\left\{ \begin{array}{l} C_{S2}-C_{S1}=C_{P}\\ \Delta d\approx a_{f}M/k \end{array}\right.$$

At this time, the offset voltage of circuit node *V_f_*, as shown in Figure 2, can be expressed as follows:

(9) $$V_{OS}\approx \frac{C_{P}G\omega_{n}^{2}d}{2C_{S}}$$

**Figure 4:**
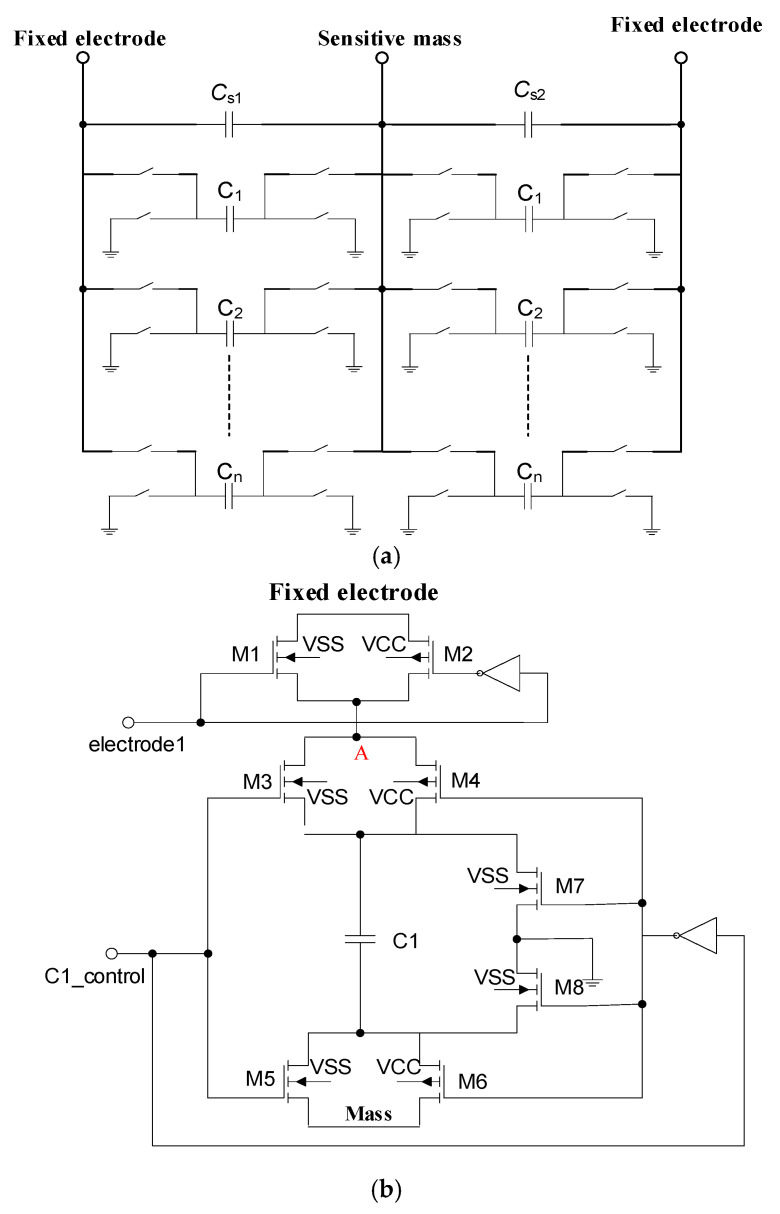
The capacitor compensation circuit: (**a**) the compensation capacitor array; (**b**) compensation capacitor array cell.

This offset voltage $V_{OS}$ causes the second harmonic distortion of the sensor output. In this work, we used a capacitor array to compensate for this. Therefore, the capacitor array *C*_1_ and array *C*_2_ are connected to *C_S_*_1_ and *C_S_*_2_ in parallel, as shown in Figure 4a. When there is no input acceleration signal, we set the following capacitance parameters to eliminate output offset of capacitive microaccelerometers. The voltage node *V_hold_* can be expressed as follows:

(10) $$C_{S1}+C_{P1}+C_{1}=C_{S2}+C_{P2}+C_{2}$$

(11) $$V_{hold}=\frac{(C_{S1}+C_{P1}+C_{1}-C_{S2}-C_{P2}-C_{2})A_{V}}{C_{F}}$$

The principle of capacitance compensation array is that the control signal adds the compensation capacitance to the relatively small one between differential capacitances. If the sensitive element does not need compensation, the two plates of the compensation capacitor are connected to the ground potential by the control signal. The capacitance compensation array module is shown in Figure 4b. When electrode 1 is high level, M1 and M2 turn on, and the fixed electrode is connected to point A, as shown in Figure 4b with red marks. At this time, when C1_control is high level, M3, M4, M5, and M6 turn on, and the compensation capacitance C1 is connected between the fixed electrode and the mass block. This can eliminate sensor misalignment by adjusting the compensation capacitance. When C1_control is low level, M7 and M8 turn on, and the plates of capacitance C1 are both connected to the ground. In the integrated capacitance compensation array, the two plates can share C1 to Cn capacitors, as shown in Figure 4a. As long as we adjust the two control signals of electrode 1 and electrode 2, we can decide whether point A is connected to the upper plate or the lower plate of capacitance. The accelerometer sensitive element with vacuum packaged silicon structure used in this study for the design, simulation, and tests is from Colibrys Company (VS9010)(Yverdon-les-Bains, Switzerland). The major parameter indicators in the sensitive element are shown in Table 1.

Capacitive accelerometers usually operate in closed-loop, which has been proven to improve the linearity, dynamic range, and bandwidth. In this work, we used this closed-loop detection circuit based on CMOS switched capacitor topology to realize the electrostatic force feedback system for microaccelerometers. The principle of capacitive microaccelerometer interface ASIC chip is shown in Figure 3. We used the modulation and demodulation method and time-sharing multiplexing detection electrode technology in the closed-loop system. This can effectively avoid the coupling and feedthrough of the voltage feedback signal to the charge pickup channel and improve the charge detection ability of the system. The whole working cycle T of the system consists of four phases: the amplifier error pickup phase (P1), the charge amplifier preparation phase (P2), the charge sampling phase (P3), and the electrostatic force closed-loop feedback phase (P4). We have highlighted the “on” switches with red marks and “off” switches with blue marks in Figure 3. We used the correlated double sampling technique in the switched capacitor circuit to reduce the 1/*f* low-frequency noise, charge injection, and clock feedthrough. We also used the PID feedback control structure to improve system stability.

The working sequence of the analog switch is shown in Figure 3a. At P1 phase, the error of the voltage includes offset voltage and low-frequency voltage noise. The amount of charge at *V_x_* can be expressed as follows:

(12) $$Q_{x}=(V_{x}-V_{S})C_{S1}+(V_{x}+V_{S})C_{S2}=(V_{n}-V_{S})C_{S1}+(V_{n}+V_{S})C_{S2}$$

At P2 phase, switch S7 is off, and the charge amplifier is at charge detection ready state, as shown in Figure 3b. At this time, the amount of charge at *V_x_* can be expressed as follows:

(13) $$Q_{x}=(V_{n}-V_{S})C_{S1}+(V_{n}+V_{S})C_{S2}+Q_{injection}+Q_{clock}$$

When switch S8 is on, the capacitor *C_C_* stores the output voltage noise of the charge amplifier, and the amount of charge at *V_y_* can be expressed as follows:

(14) $$Q_{y}=(V_{n}-\frac{Q_{injection}+Q_{clock}}{C_{F}})\cdot C_{C}$$

The driving end of mechanical sensitive capacitors *C_S_*_1_ and *C_S_*_2_ are connected to the ground. The amount of charge at *V_x_* remains constant. The total charge can be expressed as follows:

(15) $$Q_{x}=V_{n}(C_{S1}+C_{S2})+(V_{n}-V_{OUT})C_{F}=(V_{n}-V_{S})C_{S1}+(V_{n}+V_{S})C_{S2}+Q_{injection}+Q_{clock}$$

The output voltage of the charge amplifier can be expressed as follows:

(16) $$V_{out}=V_{y}=\frac{V_{S}(C_{S1}-C_{S2})}{C_{F}}+V_{n}-\frac{Q_{injection}+Q_{clock}}{C_{F}}$$

When switch S8 is off, the stored charge of capacitor *C_C_* remains constant, and we can obtain the following formula:

(17) $$(V_{y}-V_{z})\cdot C_{C}=(V_{n}-\frac{Q_{injection}+Q_{clock}}{C_{F}})\cdot C_{C}$$

(18) $$V_{y}=\frac{V_{S}(C_{S1}-C_{S2})}{C_{F}}$$

We used this CDS technique to eliminate the low-frequency 1/*f* noise of the charge amplifier, the charge injection of the analog switch S7, and the error charge caused by clock feedthrough. The output *V_y_* is held in the sampling capacitor C_H_. At P4 phase, switches S6 and S9 are off and switch S10 is on. The sample and hold voltage *V_hold_* are fed back to the mechanical sensitive element *V_x_* by a PID circuit in order to realize the feedback system of electrostatic force. The capacitance detection circuit is completely isolated from the electrostatic force feedback circuit by switches S6 and S9, so there is no coupling between the driving signal and the detecting signal in the closed-loop interface circuit. The high-order high-pass filter in the continuous time circuit is not needed in our circuit. In this way, the demodulation output error caused by the change of filter parameters is avoided.

### 2.2. Noise Analysis of Closed-Loop Micromachined Accelerometers

The mechanical noise of the sensitive structure is generated by the thermal motion of the moving mass in microaccelerometers. The Brownian thermal motion relative to the accelerometer noise can be expressed as follows:

(19) $$a_{n}^{2}=\frac{4k_{B}TB}{M}$$

In Equation (19), *k_B_* is the Boltzmann constant; *T* is the Kelvin temperature; *B* is the damping coefficient of the mass block; *M* is the mass of the mass block. The mechanical noise is mainly determined by the mass and damping coefficient. The mass of the moving mass also limits the range of the accelerometer, and a low damping coefficient will lead to a system stability problem.

**Figure 5:**
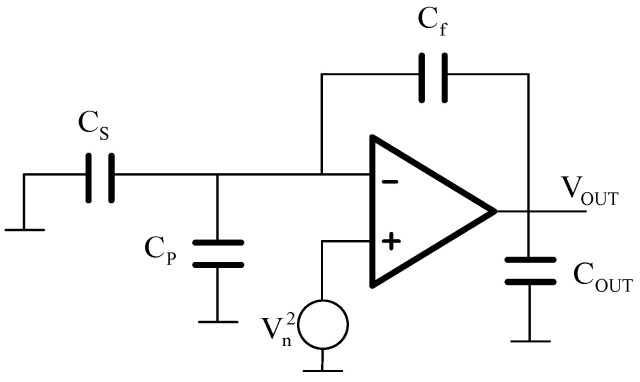
The equivalent thermal noise principle diagram of a charge-sensitive amplifier.

The noise of the front-end charge amplifier mainly includes thermal noise and 1/*f* noise. The 1/*f* noise can be suppressed to several orders of magnitude less than the thermal noise using the CDS technique in the interface circuit. Thus, the thermal noise of the charge amplifier is the main noise. The thermal noise is sampled and aliasing in the hold circuit. The noise model of the charge-sensitive amplifier in the switched capacitor circuit is shown in Figure 5. The equivalent output noise in the circuit can be expressed as follows:

(20) $$e_{Amplifier}=\sqrt{\frac{8k_{B}T(C_{S}+C_{P}+C_{F})}{3C_{F}(C_{F}+C_{out})f_{S}}}\approx \frac{1}{C_{F}}\sqrt{\frac{8k_{B}T(C_{S}+C_{P}+C_{F})}{3f_{S}}}(V/\sqrt{\mathrm{Hz}})$$

In Equation (20), *f_s_* is the sampling frequency. The equivalent noise of the charge amplifier is independent of the transistor parameters; it is only related to the capacitance of the sensitive structure, integral capacitance, and sampling frequency. We can increase the integral capacitance and sampling frequency to optimize the circuit noise. With the increase of sampling frequency, the transconductance of the charge amplifier must be increased to maintain the accuracy of signal establishment.

**Figure 6:**
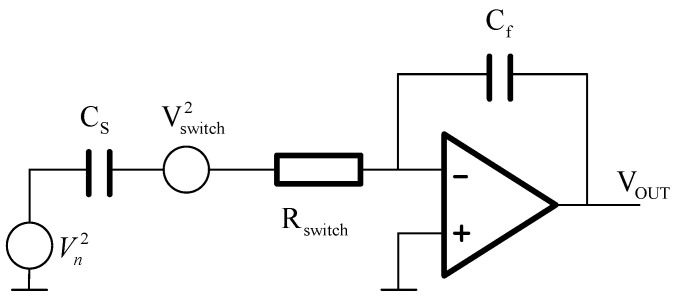
The equivalent noise model of the reference voltage source and analog switch.

Microaccelerometers can pick up the weak capacitance signal by the periodic charging and discharging of the sensitive capacitance in the sensitive structure. Therefore, any noise in the reference voltage source will worsen the signal-to-noise ratio of the microaccelerometer output. The high-frequency noise of the reference voltage will be mixed with low-frequency signal after periodic sampling. Figure 6 shows the reference voltage source noise equivalent model. The effective bandwidth of the operational amplifier limits the noise bandwidth of microaccelerometers. The equivalent output noise is as follows:

(21) $$e_{RV}=\frac{C_{S}V_{n}}{C_{F}}\sqrt{\frac{2\pi C_{F}f_{u}}{C_{T}f_{S}}}\approx \frac{V_{n}}{C_{F}}\sqrt{\frac{g_{m}C_{S}}{f_{S}}}(V/\sqrt{\mathrm{Hz}})$$

In this equation, *f_u_* is the gain bandwidth product of the operational amplifier, and *V_n_* is the noise density of the reference voltage source. The analog switch between the sensitive capacitor and the charge integrator also affects the noise of the interface circuit. The principle model of analog switching noise is shown in Figure 6. If the closed-loop charge-sensitive amplifier bandwidth is less than *f_c_*, the equivalent noise of the analog switch can be expressed as follows:

(22) $$f_{c}=\frac{1}{2\pi R_{SW}C_{S}}$$

(23) $$e_{SW}=\frac{2C_{S}}{C_{F}}\sqrt{\frac{\pi k_{B}TR_{SW}f_{u}C_{F}}{f_{S}(C_{S}+C_{P}+C_{F})}}\approx \frac{1}{C_{F}}\sqrt{\frac{2k_{B}TR_{SW}g_{m}C_{S}}{f_{S}}}(V/\sqrt{\mathrm{Hz}})$$

In this equation, *R_SW_* is the equivalent resistance of the analog switch. We can further reduce the circuit noise by reducing the equivalent resistance. However, we should make sure that the analog switch meets the requirements of *f_c_* >> *f_u_* so that the limit of the signal establishment is mainly determined by the amplifier. The noise source and theoretical expression are shown in Table 2.

### 2.3. Interface Circuit Optimization and Design

**Figure 7:**
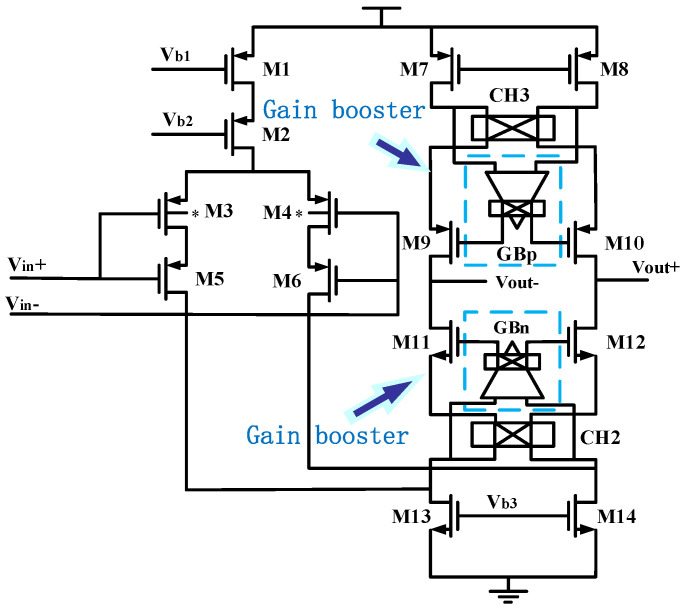
The diagram of input-stage operational amplifier in the pre-stage detection circuit.

The offset voltage of the operational amplifier used in the PID feedback control circuit will be directly fed back to the accelerometer output. The misalignment temperature coefficient will also become the key component of the microaccelerometer temperature coefficient. As the output port of microaccelerometers, the output swing of the PID feedback control circuit will affect the dynamic range of the sensor output. The open-loop gain of the operational amplifier will affect the harmonic distortion and linearity of microaccelerometers. Therefore, we adopted a three-stage operational amplifier topology and a two-stage capacitor compensation. We also adopted the fully differential topology to suppress the offset and reduce the temperature coefficient. The input-stage operational amplifier circuit structure is shown in Figure 7.

**Figure 8:**
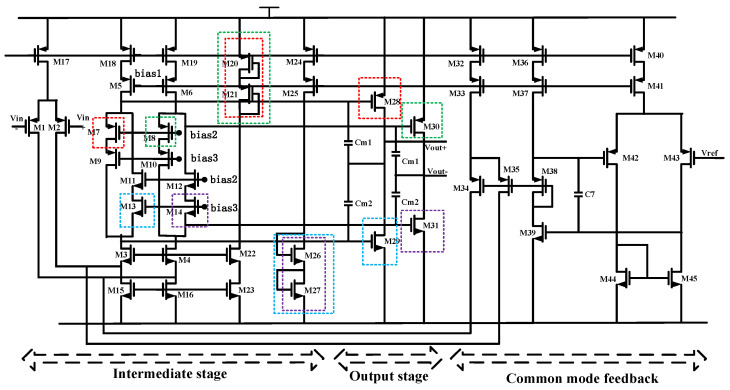
Circuit diagram of the intermediate stage and the output stage.

We considered the 1/*f* noise and *KT/C* noise in the interface ASIC based on the weak charge signal of microaccelerometers. We used the common-gate M3 and M4 transistors, which were connected to the virtual location, to reduce the substrate bias effect of input-stage transistors in our design. We also used a fully differential folded cascode structure with common-mode feedback in the input-stage circuit. In order to suppress circuit noise and achieve higher gain accuracy, we used the gain booster technique to improve DC gain in the PID feedback and CSA. The circuit and layout have been designed to satisfy the size and symmetry. The circuit diagram of the intermediate stage and the output stage is shown in Figure 8. The intermediate stage adopts folded cascode structure to improve the gain. The output stage adopts class-AB fashion to achieve rail-to-rail output. In order to save power, the class-AB mesh structure is incorporated into the output branch of the intermediate stage. The bias condition of the class-AB stage is determined by four translinear loops, which are marked in color in Figure 8. One of the translinear loop M20, M21, M7 and M28 can be expressed as follows:

(24) $$V_{GS20}+V_{GS21}=V_{GS7}+V_{GS28}$$

According to the current and voltage equation of saturation region,

(25) $$V_{GS}=V_{TH}+\sqrt{\frac{2I_{DS}}{\mu_{p}C_{ox}\frac{W}{L}}}$$

Finally, we can obtain Equation (26). To maintain the same current-to-dimension ratio expressed by Equation (25), the transistors within the translinear loops are all well-matched in the layout.

(26) $$\sqrt{I_{DS20}}\left(\frac{1}{\sqrt{{\left(\frac{W}{L}\right)}_{20}}}+\frac{1}{\sqrt{{\left(\frac{W}{L}\right)}_{21}}}\right)=\sqrt{\frac{I_{DS7}}{{\left(\frac{W}{L}\right)}_{7}}}+\sqrt{\frac{I_{DS28}}{{\left(\frac{W}{L}\right)}_{28}}}$$

**Figure 9:**
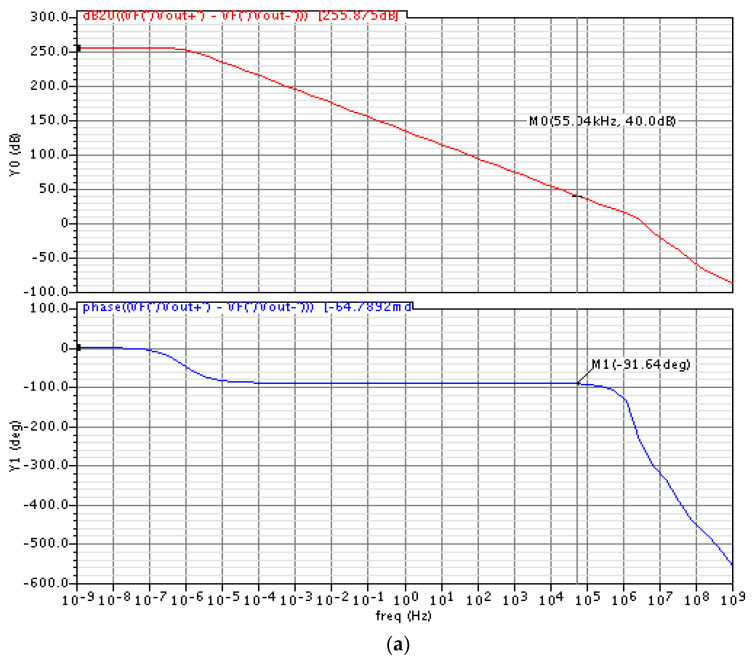

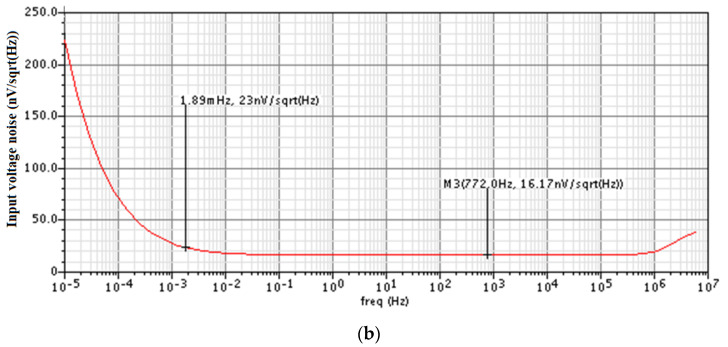
Simulation of the interface circuit: (**a**) amplitude frequency characteristic simulation in Cadence; (**b**) noise characteristic simulation in Cadence.

We simulated the amplitude frequency characteristics and noise characteristic of the interface circuit, as shown in Figure 9. The circuit can achieve a DC gain of 256 dB and a bandwidth of 55.34 kHz at 40 dB closed-loop gain. The noise of the interface circuit is about 16.17 nV/Hz^1/2^. The noise corner frequency is in the order of mHz.

## 3. Results and Discussion

**Figure 10:**
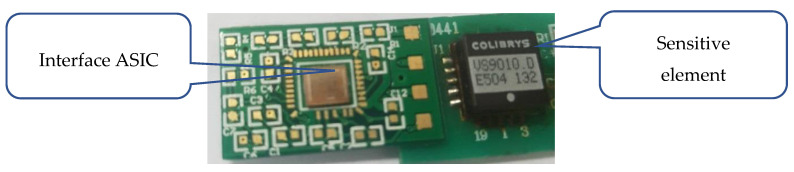
PCB photograph of the interface chip and sensors.

**Figure 11:**
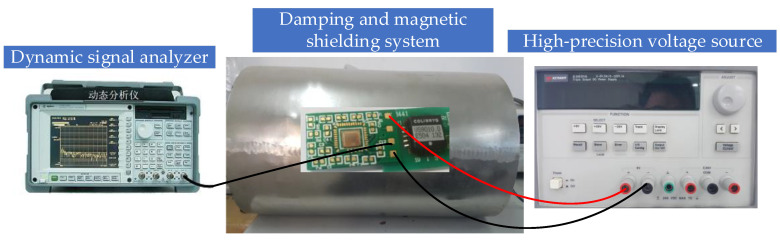
The microaccelerometer measurement system.

To verify the analysis presented in the previous sections, the interface circuit chip was designed in a standard 0.35 μm CMOS process. The printed circuit board (PCB) photograph of a microaccelerometer is shown in Figure 10. The interface circuit chip, which had 40 PAD pins for the chip test, is also shown in Figure 10. The pins on the interface ASIC chip were connected with silicon aluminum wire by a welding machine. The active area of the chip was 3.2 mm × 3 mm. We verified the function of the interface circuit before testing the performance of the microaccelerometer. The 5 V power supply was supported by Agilent E3631 (Agilent Technologies Inc., Santa Clara, CA, USA). The Agilent 35670A spectrum analyzer (Agilent Technologies Inc., Santa Clara, CA, USA) was used for the noise test of the interface circuit. The microaccelerometer measurement system is shown in Figure 11. The accelerometer was placed in an environment of shock absorption and magnetic shielding.

**Figure 12:**
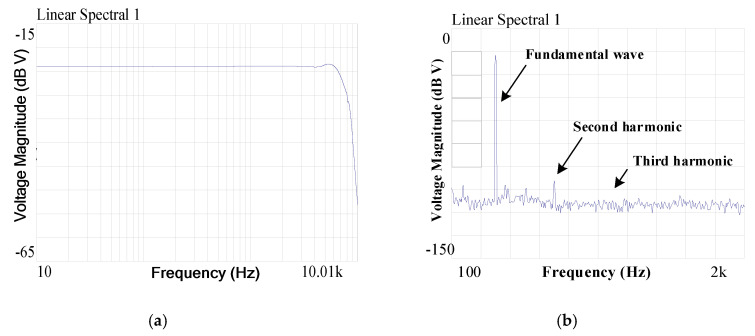
Test of the microaccelerometer system: (**a**) frequency response testing curve; (**b**) noise spectrum testing curve.

The frequency response and noise spectrum testing of the closed-loop microaccelerometer are shown in Figure 12. The low-frequency environment vibrations were still visible through a simple mass spring shock absorption system. A simple electromagnetic shielding was used to reduce the line-frequency harmonics. The power dissipation of the interface circuit chip was 7.7 mW at a sampling frequency of 10 kHz. We set the frequency to be much higher than the Nyquist sampling rate, which can prevent noise aliasing.

**Figure 13:**
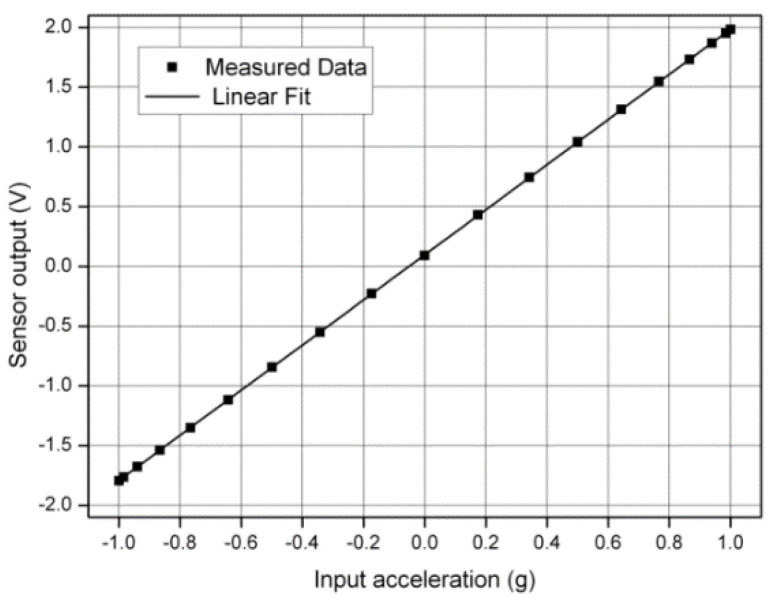
The nonlinearity test of the closed-loop microaccelerometer.

The resulting SNR was 110 dB when referred to ±1.2 g full-scale (FS) DC acceleration. The input signal and clock signal were supplied by a Tektronix AFG3102 (Tek Technology Company, Shanghai, China) function signal generator. Nonlinearity of the closed-loop microaccelerometer was measured using a rate table, as shown in Figure 13. We optimized the noise of the analog switches and CSA in the interface circuit. The interface circuit had a dynamic range of 120 dB. The average noise floor in low-frequency range was less than −130 dBV, as shown in Figure 12b. The test results showed that the interface circuit of the microaccelerometer allowed it to achieve sub-μg output noise density. The full test of the microaccelerometer system is shown in Table 3.

## 4. Conclusions

In this work, we propose a switched capacitor interface circuit for microaccelerometers. In this system, the charge-sensitive time sequence is separated from the electrostatic feedback time sequence in order to avoid the coupling effect. The noise characteristics of the interface circuit was analyzed and optimized. The layout design and engineering batch chip were fabricated by a standard 0.35 μm CMOS process. The test results of a closed-loop microaccelerometer showed that the dynamic range of the interface circuit could achieve 120 dB and an average noise floor could achieve −130 dBV at a low-frequency range. The resolution of the microaccelerometer could reach 0.5 μg/Hz^1/2^ (@200 Hz); the bias stability of the microaccelerometer was 50 μg, and the nonlinearity was 0.15% FS at ±1.2 g.

In Table 4, we compare the performance attained in this work with previously reported accelerometers. There are three kinds of circuit structures: hybrid switched capacitor (S-C), closed-loop continuous-time, and closed-loop switched capacitor. The microaccelerometer system we have proposed can achieve better performance than most of the reported accelerometers. This work is advantageous with regard to noise floor compared to [14,24,25]. Although the work presented in [16] also has an excellent noise floor, it has higher power dissipation than our work.

## Figures and Tables

**Table 1:** Parameters of the sensitive element.

Parameters	Value
Sensitivity	200 ± 2 mV/g
Full-scale range	±10 g
Bandwidth (−3 dB)	0 to ≥2.4 kHz
Resonant frequency (ω_0_)	2 kHz
Nonlinearity	<1% FS
Noise spectral density in band	25 μV/$\sqrt{\mathrm{Hz}}$

**Table 2:** The noise source of the microaccelerometer system.

The Noise Source	Theoretical Expression
Thermal noise of the sensitive element	$a_{n}^{2}=\frac{4k_{B}TB}{M}$
The equivalent noise of the pre-stage charge-sensitive amplifier (CSA)	$e_{Amplifier}=\frac{1}{C_{F}}\sqrt{\frac{8k_{B}T(C_{S}+C_{P}+C_{F})}{3f_{S}}}$
The equivalent noise of the reference voltage	$e_{RV}\approx \frac{V_{n}}{C_{F}}\sqrt{\frac{g_{m}C_{S}}{f_{S}}}$
The equivalent noise of the switch	$e_{SW}=\frac{1}{C_{F}}\sqrt{\frac{2k_{B}TR_{SW}g_{m}C_{S}}{f_{S}}}$

**Table 3:** Performance of the microaccelerometer system.

MEMS Sensitive Element and Interface Circuit	
Sensitivity (mV/g)	1200
Input range (g)	±1.2
Bandwidth (Hz)	1000
Power dissipation (mW)	7.7
Nonlinearity	0.15%
Bias instability (μg)	50 μg
Noise floor (μg/Hz^1/2^)	0.5 (@200 Hz)

**Table 4:** Comparison of this work with other microaccelerometers.

Parameters	[24]	[16]	[14]	[25]	This Work
Circuit structure	Hybrid S-C	Closed-loop continuous-time	Closed-loop S-C	Closed-loop S-C	Closed-loop S-C
Process (μm)	0.35	0.7	0.5	0.6	0.35
Sensitivity(V/g)	0.495	2.267	NA	0.373	1.2
Noise floor (μg/Hz^1/2^)	2(@200 Hz)	0.3 (@300 Hz)	4 (@500 Hz)	1.15 (@300 Hz)	0.5 (@200 Hz)
Power (mW)	3.6	85.8	4.5	12	7.7
Supply/Range	3.6 V/±1.15 g	5 V/±1.5 g	3 V/NA	9 V/±11 g	5 V/±1.2 g

## References

[1] Wang C., Chen F., Wang Y., Sadeghpour S., Wang C.X. (2020). Micromachined Accelerometers with Sub-mu g/root Hz Noise Floor: A Review. Sensors.

[2] Yuan Z.Y., Han E.C., Meng F.L., Zuo K.Y. (2020). Detection and Identification of Volatile Organic Compounds based on Temperature-Modulated ZnO Sensors. Instrum. Meas. IEEE Trans..

[3] Soen J., Voda A., Condemine C. (2019). Implementation of a CMOS/MEMS Accelerometer with ASIC Processes. Micromachines.

[4] Chen D.L., Yin L., Fu Q., Zhang W.B., Wang Y.H. (2020). A Straightforward Approach for Synthesizing Electromechanical Sigma-Delta MEMS Accelerometers. Sensors.

[5] Kar S.K., Chatterjee P., Mukherjee B., Swamy K.B.M.M., Sen S. (2018). A Differential Output Interfacing ASIC for Integrated Capacitive Sensors. IEEE Trans. Instrum. Meas..

[6] Li X.Y., Hu J.P., Liu X.W. (2018). A High-Performance Digital Interface Circuit for a High-Q Micro-Electromechanical System Accelerometer. Micromachines.

[7] Gomez J.M., Bota S.A., Marco S. (2020). Electrical equivalent modeling of MEMS differential capacitive accelerometer. Microelectron. J..

[8] Petkov V.P., Balachandran G.K., Beintner J. (2014). A Fully Differential Charge-Balanced Accelerometer for Electronic Stability Control. IEEE J. Solid-State Circuits.

[9] Li H.W., Zhai Y.X., Tao Z., Gui Y.X., Tan X. (2020). Thermal Drift Investigation of an SOI-Based MEMS Capacitive Sensor with an Asymmetric Structure. Sensors.

[10] Wang Y.H., Fu Q., Zhang Y.F., Liu X.W. (2020). A Digital Closed-Loop Sense MEMS Disk Resonator Gyroscope Circuit Design Based on Integrated Analog Front-end. Sensors.

[11] Wu P.C., Liu B.D., Yeh C.Y. Design of a 0.6-V 0.2-mW CMOS MEMS Accelerometer. Proceedings of the 2015 IEEE International Conference on Consumer Electronics.

[12] Xu H.L., Liu X.W., Yin L. (2015). A Closed-Loop Sigma Delta Interface for a High-Q Micromechanical Capacitive Accelerometer with 200 ng/root Hz Input Noise Density. IEEE J. Solid-State Circuits.

[13] Chen D.L., Liu X.W., Yin L., Wang Y.H., Shi Z.H., Zhang G.R. (2018). A Sigma Delta Closed-Loop Interface for a MEMS Accelerometer with Digital Built-In Self-Test Function. Micromachines.

[14] Amini B.V., Abdolvand R., Ayazi F. (2006). A 4.5-mW closed-loop micro-gravity CMOS SOI accelerometer. IEEE J. Solid-State Circuits.

[15] Aaltonen L., Rahikkala P., Saukoski M., Halonen K. (2009). High-resolution continuous time interface for micromachined capacitive accelerometer. Int. J. Circuit Theory Appl..

[16] Aaltonen L., Halonen K. (2009). Continuous-time interface for a micromachined capacitive accelerometer with NEA of 4 g and bandwidth of 300 H. Sens. Actuators A Phys..

[17] Chen D.L., Yin L., Fu Q., Liu X.W. (2020). Measuring and calibrating of the parasitic mismatch in MEMS accelerometer based on harmonic distortion self-test. Sens. Actuators A Phys..

[18] Zhong L.J., Yang J., Xu D.L., Lai X.Q. (2020). Bandwidth-Enhanced Oversampling Successive Approximation Readout Technique for Low-Noise Power-Efficient MEMS Capacitive Accelerometer. IEEE J. Solid-State Circuits.

[19] Wang Y.M., Chan P.K., Li H.K.H. (2015). A Low-Power Highly-Sensitive Capacitive Accelerometer IC Using Auto-Zero Time-Multiplexed Differential Technique. IEEE Sens. J..

[20] Liu D.D., Liu H.F., Liu J.Q. (2020). Gradient Method for Alleviating Bonding-Induced Warpage in a High-Precision Capacitive MEMS Accelerometer. Sensors.

[21] Song Z., Sun T., Wu J. (2014). System-Level Simulation and Implementation for a High Q Capacitive Accelerometer with PD Feedback Compensation. Microsyst. Technol..

[22] Chen F., Zhao Y., Wang J.C., Zou H.S., Kraft M., Li X.X. (2018). A Single-Side Fabricated Triaxis (111)-Silicon Microaccelerometer with Electromechanical Sigma–Delta Modulation. IEEE J. Solid-State Circuits.

[23] Akita I., Okazawa T., Kurui Y., Fujimoto A., Asano T. (2020). A Feedforward Noise Reduction Technique in Capacitive MEMS Accelerometer Analog Front-End for Ultra-Low-Power IoT Applications. IEEE J. Solid-State Circuits.

[24] Yucetas M., Pulkkinen M., Kalanti A. (2012). A high-resolution accelerometer with electrostatic damping and improved supply sensitivity. IEEE J. Sens..

[25] Pastre M., Kayal M., Schmid H., Huber A. A 300Hz 19b DR capacitive accelerometer based on a versatile front end in a 5th-order ΔΣ loop. Proceedings of the 2009 Proceedings of ESSCIRC.

